# Understanding women’s perspectives on breast cancer is essential for cancer control: knowledge, risk awareness, and care-seeking in Mwanza, Tanzania

**DOI:** 10.1186/s12889-020-09010-y

**Published:** 2020-06-15

**Authors:** Christina A. Chao, Liuye Huang, Kala Visvanathan, Kisa Mwakatobe, Nestory Masalu, Anne F. Rositch

**Affiliations:** 1 Department of Epidemiology, Johns Hopkins Bloomberg School of Public Health, Baltimore, Maryland USA; 2 Department of Oncology, Johns Hopkins School of Medicine, Baltimore, Maryland USA; 3 Tanzania Breast Cancer Foundation, Dar es Salaam, Tanzania; 4 Department of Oncology, Bugando Medical Centre, Mwanza, Tanzania

**Keywords:** Breast Cancer, Breast health, Tanzania, Care-seeking behavior, Knowledge-attitudes-practices survey, BCAM

## Abstract

**Background:**

Breast Cancer is the most common cancer in women worldwide. Since 2008, Mwanza, Tanzania, has worked to provide comprehensive cancer services through its Zonal consultant hospital. New national guidelines focused on clinical breast exam requires that women be aware of and seek care for breast concerns. Therefore, this study aims to understand breast cancer awareness in Mwanza and describe women-level barriers, care-seeking behavior, and perspectives on breast cancer.

**Methods:**

A community-based survey was administered to conveniently sampled women aged 30 and older to assess women’s perspectives on breast cancer and care-seeking behavior.

**Results:**

Among 1129 women with a median age of 37 (IQR: 31–44) years, 73% have heard of cancer and 10% have received breast health education. Women self-evaluated their knowledge of breast cancer (from 1-none to 10-extremely knowledgeable) with a median response of 3 (IQR: 1–4). Only 14% felt they knew any signs or symptoms of breast cancer. Encouragingly, 56% of women were fairly-to-very confident they would notice changes in their breasts, with 24% of women practicing self-breast examination and 21% reporting they had received a past breast exam. Overall, 74% said they would be somewhat-to-very likely to seek care if they noticed breast changes, with 96% noting severity of symptoms as a motivator. However, fear of losing a breast (40%) and fear of a poor diagnosis (38%) were most frequent barriers to care seeking. In assessing knowledge of risk factors, about 50% of women did not know any risk factors for breast cancer whereas 42% of women believed long term contraceptive use a risk factor. However, 37% and 35% of women did not think that family history or being older were risk factors, respectively.

**Conclusions:**

The success of efforts to improve early diagnosis in a setting without population-based screening depends on women being aware of breast cancer signs and symptoms, risks, and ultimately seeking care for breast concerns. Fortunately, most women said they would seek care if they noticed a change in their breasts, but the low levels of cancer knowledge, symptoms, and common risk factors highlight the need for targeted community education and awareness campaigns.

## Background

Breast Cancer is the most common cancer in women worldwide. In high income countries, high visibility public health efforts and advocacy groups have made breast cancer screening programs and education campaigns successful. In low- and middle- income countries (LMICs) incidence and mortality is on the rise and breast cancer is the most common cause of cancer deaths among women [1]. In Tanzania, breast cancer incidence is projected to increase 82% by 2030 [2]. To combat this, and other cancers, Tanzania has developed guidelines for cancer control and have made efforts to equip major specialty hospitals with diagnostic tools and expanded oncology departments [2, 3].

In Mwanza, Tanzania, Bugando Medical Centre (BMC) serves as the area’s Zonal consultant hospital, with a catchment area of the entire lake region [3] serving over 15 million people [4]. In the past ten years, BMC has worked to create, staff and train advanced oncology, surgery, and pathology departments to provide cancer diagnostic, treatment, and radiation services [2]. While these services are available, they are underutilized by the general community as women often present to the hospital as late stage or palliative breast cancer cases [5–7]. Late stage presentation--an estimated 80% of all cases--limits women’s options for care and patients cannot take advantage of the full range of treatment options available to them [2]. About half of all women diagnosed with breast cancer will die from the disease [2]. This trend however is not just common to Tanzania, but to many other LMICs as mortality rates are generally higher than those of high- income countries (HICs) despite currently lower incidence rates [8, 9].

Specifically, in Mwanza, research into the subject of breast cancer, and all cancers, has been limited. Pathology studies have estimated an increase in breast cancer and have shown a higher prevalence of aggressive breast cancers [6, 7, 10]. Studies looking into biological features of breast cancer have taken priority for research as these are most helpful in building and arming cancer centers and influence drug and treatment stock. Missing from the literature, however, are data on women’s’ knowledge, attitudes, and practices surrounding breast care and breast cancer. [6, 7, 11] Poor prognosis at diagnosis has been shown to be associated with lower breast cancer awareness and not tumor characteristics alone [9]. To understand breast cancer awareness in Mwanza, Tanzania and address women-level barriers to cancer control, we conducted an extensive community-based Knowledge, Attitudes and Practices survey. As Tanzania continues to develop policies and clinical programs to combat the increasing breast cancer incidence and mortality, understanding women’s perspectives on breast cancer and care-seeking are fundamental to successful cancer control through early detection [12].

## Methods

A cross-sectional Knowledge, Attitudes and Practices (KAP) survey was carried out in the Mwanza region of Tanzania. This survey was conducted as part of a three phase, adaptive implementation science study, which employed a novel “A.C.T.” framework to Assess the context, Couple strategies to fit the context via stakeholder engagement, and Test the identified strategies to improve breast cancer care and control [13]. The KAP survey, which was part of the assessment phase, was administered in Nyamagana, Ilemela and Sengerema, which are within the catchment areas of Bugando Medical Centre, the zonal hospital with cancer care capabilities. A convenience sample of women aged 30 or older were recruited by trained, Tanzanian female research staff in public areas such as bus stops, train stops, and open markets in order to enroll general community women. Once eligibility criteria were confirmed, administrators consented participants per IRB requirements and then administered the survey. Consent and the survey were administered in Swahili to ensure widespread understanding. This study was approved by National Institutes for Medical Research, Tanzania (NIMR), the Catholic University of Health and Allied Sciences-Bugando Medical Centre (CUHAS-BMC), and from Johns Hopkins Bloomberg School of Public Health (JHSPH).

### Survey development and administration

The core of the KAP survey was based on the Breast Cancer Awareness Measures (BCAM) [14] developed by Cancer Research UK [15]. This series of questionnaires were developed to specifically assess cancer awareness in the general population and are tailored to the specific cancer [14]. In the field, the BCAM has demonstrated high construct validity as well as readability [15–17], and has successfully been used in East Africa [18]. Additional questions were added to the subset of selected BCAM questions in order to meet the study objectives and best adapt to the local context. Our KAP survey included sections adapted from the BCAM on self-reported cancer awareness, familiarity of cancer and knowledge of symptoms, confidence, skills and behavior about changes in the breasts, previous contact with the health system regarding breast health, barriers to care, knowledge of risk factors. Questions about sociodemographics, breast cancer burden and care specifically in Tanzania, and a knowledge self-assessment were added by the study investigators. Knowledge of breast cancer symptoms was assessed twice, once by asking if women knew any symptoms and if yes, then recording those they recalled. Next, all women were read a list of symptoms and asked to indicate those they recognized as symptoms.

To ensure appropriate use of language and translation, the survey was fully developed in English then translated to Swahili using a native Tanzanian Swahili speaker. The translated survey was then back translated into English by a different native Tanzanian Swahili speaker to check for consistency. Any inconsistencies were worked out between the Principal investigator (A.F. Rositch) and the two translators to maintain question integrity. The survey was programmed into Qualtrics Survey Software (Qualtrics, Provo, UT) and downloaded onto password protected tablets to be utilized in the field. Experienced survey administrators were trained on proper use of tablets and Qualtrics survey, consent procedures, and consistent language and approach to asking survey questions. The survey was pilot tested prior to roll out for training purposes and to ensure a culturally appropriate language and understanding. Survey responses were entered directly into Qualtrics in real-time by the interviewer and then uploaded to the secure server at a later time. This allowed women to be surveyed anywhere, while increasing data security and minimizing data loss. Data was output directly from Qualtrics, where it was quality checked, translated into English, and free text was cleaned and categorized (if applicable).

### Data and analysis

We assessed knowledge of breast cancer through a series of questions about breast cancer symptoms, survival, risk factors or statistics. Risk Awareness was assessed through questions that specifically asked the woman’s perspective on her own body, chances of developing breast cancer and familial history of breast cancer. Attitudes and practices around breast cancer and care-seeking were assessed through questions pertaining to behaviors, potential barriers, experience with the healthcare system and likelihood of care seeking. Descriptive statistics (frequencies, proportions, and mean/medians) were used to describe the population demographics, basic breast cancer knowledge, and risk factor awareness. Data on barriers and motivators of care-seeking are visually displayed.

Analysis of Variance (ANOVA) was used to estimate and compare mean knowledge scores between demographic, risk, and care-seeking variables and both self-reported knowledge and a built knowledge score. The Tukey Test was used to determine subgroups with significantly different knowledge levels, with alpha = 0.05. Self-reported knowledge was measured directly on the survey by asking women to rate their knowledge of breast cancer on a scale of 1–10. The built knowledge score was calculated based on knowledge of risk factors and knowledge of symptoms or signs, with a maximum score of 12.5. For each known risk factor (from Table 3), 1 point was awarded to the score. Women were also awarded 1 point if they simply indicated that they knew signs and symptoms of breast cancer (from Table 3); they earned 0.25 for each specific sign or symptom they free listed or indicated knowing when read off to them. Women were also awarded 1 point for understanding the importance of early detection (from Table 2). The self-reported score and the knowledge-built score were both converted to percentages (0–100%) for analysis and comparability. Data were analyzed using Stata 12.1 (StataCorp, College Station, TX).

## Results

A total of 1129 women were enrolled and completed the survey. The median age of women was 37 (IQR 31–45), and the majority of women were married (62.7%) and identified as Christian (73.6%) (Table 1). Access to transportation was limited with only 8.4% of women indicating they had access to a personal car or van and 6.9% indicating they had access to a motorcycle. The majority of women completed some formal education (65.3%; equivalent to primary school in the United States) and very few (7.0%) had school at the university/college level or beyond. Only 21.4% of women reported having health insurance and almost all women reported visiting a traditional healer in the past (85.5%).

**Table 1:** Demographics of 1129 participants in the KAP survey in the Lake Zone of Tanzania

Age, median (IQR)	37 (31–45)
Age Categories	
30–39	635 (56.2%)
40–49	293 (26.0%)
50–59	163 (14.4%)
60–69	28 (2.5%)
70+	10 (0.9%)
Region	
Urban	304 (26.9%)
Peri Urban	419 (37.1%)
Rural	406 (36.0%)
Marriage	
Married	708 (62.7%)
Not married	330 (29.2%)
Divorced or Separated	91 (8.1%)
Religion	
Christianity	831 (73.6%)
Muslim	297 (26.3%)
Other	1 (0.1%)
Living arrangement	
Rent from NHC	34 (3.0%)
Rent from private landlord	774 (68.5%)
Own outright or have a mortgage	309 (27.4%)
Other or Don’t know	12 (1.1%)
Transportation	
No transportation	907 (80.3%)
Access to Bicycle	49 (4.4%)
Access to Motorcycle	78 (6.9%)
Access to car or Van	95 (8.4%)
Occupation	
Peasant	209 (18.5%)
Business	748 (66.3%)
Manual labor	88 (7.8%)
Professional	58 (5.1%)
Other	26 (2.3%)
Highest education received	
No schooling or Don’t know	143 (12.7%)
Up to Class	737 (65.3%)
Up to Form	170 (15.0%)
University and Post University	79 (7.0%)
Health Insurance	
Yes	242 (21.4%)
No or Don’t know	887 (78.6%)
Ever visited a Traditional Healer	
Yes	965 (85.5%)
No	164 (14.5%)

Most women indicated that they had heard of cancer (73.9%), and 10.4% reported they had received previous breast cancer education (Table 2). A quarter of women said they know someone (family or otherwise) who has or had breast cancer, and 2.7% reported they had been diagnosed with breast cancer themselves. On a scale from 1 to 10, women self-rated their knowledge of breast cancer to be very low (Median 3; IQR: 1–4). Of the 155 women who said that they knew breast cancer symptoms (13.7%), a lump or thickening in the breast (69.8%) or under the armpit (75.5%) were the most common free-listed symptoms. Other top responses included pain in the breasts or armpit (49.7%), nipple rash (46.4%) and discharge or bleeding from the nipple (43.9%). The most commonly known symptoms among all women when they were read-off were, similarly, a lump or thickening in the breast (36.4%) or under the armpit (34.8%), pain in the breasts or armpit (34.2%) and discharge or bleeding from the nipple (32.6%).

**Table 2:** Breast Cancer knowledge and risk awareness of women in the community knowledge assessment

Response		N (%)	
Have you ever heard of cancer?			
Yes		834 (73.9%)	
No		295 (26.1%)	
Have you ever received previous education about breast health or breast cancer?			
Yes		119 (10.5%)	
No		1010 (89.5%)	
Do you know anyone, family or otherwise, who has been diagnosed with breast cancer?			
Yes		293 (26.0%)	
No		836 (74.0%)	
Have you ever been told that you have breast cancer?			
Yes		30 (2.7%)	
No		1099 (97.3%)	
On a scale from 1 to 10 how would you rate your knowledge of breast cancer?			
median (IQR)		3 (1–4)	
Do you know any of the warning signs or symptoms of breast cancer?			
Yes		155 (13.7%)	
No		974 (86.3%)	
**Symptoms**	Free listed^*^	Read off^**^	
	*N* = 155	*N* = 155	*N* = 1129
Change in position of the nipple	32 (20.6%)	89 (57.4%)	291 (25.8%)
Puckering or Dimpling of the breast skin	35 (22.6%)	91 (58.7%)	308 (27.3%)
Nipple rash	72 (46.4%)	116 (74.8%)	360 (31.9%)
A lump or thickening under the armpit	117 (75.5%)	122 (78.7%)	393 (34.8%)
Pulling in of the nipple	58 (37.4%)	106 (68.4%)	347 (30.7%)
Discharge or bleeding from the nipple	68 (43.9%)	111 (71.6%)	368 (32.6%)
Redness of the Breast skin	36 (23.2%)	87 (56.1%)	322 (28.5%)
Changes in the shape of the breast or nipple	35 (22.6%)	89 (57.4%)	336 (29.8%)
Pain in one of the breasts or armpits	77 (49.7%)	111 (71.6%)	386 (34.2%)
A lump or thickening in the breast	108 (69.8%)	124 (80.0%)	411 (36.4%)
Changes in the size of the breast or nipple	31 (20.6%)	88 (56.8%)	307 (27.2%)

^*^Free listed: If women indicated that they knew any of the signs or symptoms of breast cancer they were then asked to verbally list the ones they knew. These were captured and recorded. ^**^Read off: Once women were done free listing OR if they did not know any signs or symptoms of breast cancer, they were then read this list of signs and symptoms and asked to indicate which ones they believed were true

Risk factors awareness was assessed by asking women to rate their agreement on a series of statements (Table 3). Some risk factors got lukewarm responses, such as “Being overweight (BMI over 25) can increase the chance of getting breast cancer” and “Mutations in your genes (BRAC1 or BRAC2) can increase the chance of getting breast cancer” with about 50.0% of women indicating that they were “not sure” and about 25.0% of women either agreeing or disagreeing. Notably, 41.6% of women agree that long term contraceptive use is a risk factor for breast cancer. Women did not think that family history or being older were risk factors, with 15.1 and 14.6% agreement, respectively. When women were asked to list any other risk factors they knew or believed to be causes of breast cancer, responding women (*n* = 84) often referred to food, clothing, and traditional healers or drugs as being risk factors. Examples of food-related responses included drinking processed juices or soda, eating foods from plastic containers, excessive alcohol consumption and diet in general. Pertaining to clothing, women believed that wearing dirty or wet clothes, tight bras, and secondhand clothes were risk factors. Additionally, storing money or cell phones in the bra, and wearing black bras in the sun were listed as risk factors. Vaccinations by a traditional healer, using traditional medicine for a long time, using drugs without a Medical Doctors approval, and using drugs to enlarge breasts were also believed to be risk factors for breast cancer. Most women couldn’t gauge their own chances of developing breast cancer, with 53.5% of women reporting they were unsure of their lifetime risk of breast cancer. Fortunately, the majority of women agreed that early detection is important (72.3%), it is possible to treat breast cancer (69.7%), and that treatment is available to them in Mwanza (58.3%).

**Table 3:** Knowledge of breast cancer risk factors of KAP participants

Risk Factors Response N (%)	
Being older can increase the chance of getting breast cancer	
Disagree	399 (35.3%)
Not Sure	565 (50.1%)
Agree	165 (14.6%)
Not breastfeeding can increase the chance of getting breast cancer	
Disagree	211 (18.7%)
Not Sure	600 (53.1%)
Agree	318 (28.2%)
Having a close relative with breast cancer can increase the chance of getting breast cancer	
Disagree	414 (36.7%)
Not Sure	544 (48.2%)
Agree	171 (15.1%)
Long term hormone contraceptive use can increase the chance of getting breast cancer	
Disagree	145 (12.9%)
Not Sure	514 (45.5%)
Agree	470 (41.6%)
Being overweight (BMI over 25) can increase the chance of getting breast cancer	
Disagree	244 (21.6%)
Not Sure	650 (57.6%)
Agree	235 (20.8%)
Do you know any other risk factors?	Yes 84 (7.4%)
If Yes, Of or pertaining to food or drink^†^	34 (40.5%)
If Yes, Traditional Healer/Traditional drugs^‡^	16 (19.0%)
If Yes, Of or pertaining to Bras and Clothes^§^	12 (14.3%)
If Yes, Other	22 (26.2%)
What do you think your own chances are to develop breast cancer in your lifetime?	
Likely	145 (12.8%)
Moderate	273 (24.2%)
Unlikely	107 (9.5%)
Don’t know	604 (53.5%)
Early detection is important to surviving breast cancer	
Disagree	14 (1.2%)
Not Sure	299 (26.5%)
Agree	816 (72.3%)
It is possible to treat breast cancer	
Disagree	43 (3.8%)
Not Sure	299 (26.5%)
Agree	787 (69.7%)
There is treatment available for you to treat breast cancer in Mwanza?	
Disagree	63 (5.6%)
Not Sure	408 (36.1%)
Agree	658 (58.3%)

^†^Examples include: Food in general, Drinking Juice or Soda, Eating food from plastic containers, excessive alcohol consumption. ^‡^Examples include: Being vaccinated by a traditional healer, using traditional medicine for a long time, using drugs without a Medical Doctors approval, using drugs to enlarge breasts. ^§^Examples include: Wearing secondhand clothes, wearing dirty or wet clothes, wearing a tight bra, wearing a black bra in the hot sun, keeping money in the bra

Care-seeking practices around breast health were very limited, with 21.7% of participants reporting that they have ever been checked for any type of cancer (Table 4). Specifically, 12.8% reported having ever had a clinical breast exam (CBE) and 5.2% reported either having a mammogram or ultrasound. Only 23.6% of women reported self-examining their breasts before, although most women indicated they were very confident (16.3%) or fairly confident (39.4%) they would notice a change in their breast. If women noticed a change in their breasts, the majority of women said they were very (25.3%) or somewhat likely (47.2%) to seek care, and that the first place they would go is the district hospital (66.1%) as opposed to a community health clinic (31.7%) or traditional healer (2.2%).

**Table 4:** Breast cancer practices and care seeking behavior of KAP participants

Response	N (%)
Have you ever been checked for cancer?	
Yes	245 (21.7%)
No	884 (78.3%)
Have you ever had a clinical breast exam?	
Yes	144 (12.8%)
No	985 (87.2%)
Have you ever had any other type of breast exam (mammogram or ultrasound?)	
Yes	59 (5.2%)
No	1070 (94.8%)
Have you ever examined your breasts yourself to feel for any lumps or cancer?	
Yes	266 (23.6%)
No	863 (76.4%)
If yes, how often do you check your breasts?	
At least once a week	76 (28.6%)
At least once a month	23 (8.6%)
At least once every 6 months	13 (4.9%)
Rarely or Never	154 (57.9%)
Are you confident you would notice a change in your breasts?	
Very Confident	184 (16.3%)
Fairly Confident	445 (39.4%)
Slightly Confident	154 (13.7%)
Not at all Confident	296 (26.2%)
Don’t know	50 (4.4%)
If you noticed changes in your breasts how likely are you to seek care?	
Very likely	286 (25.3%)
Somewhat likely	533 (47.2%)
Not Sure	101 (9.0%)
Somewhat unlikely	79 (7.0%)
Very unlikely	130 (11.5%)
If you had a question about your breast or noticed a lump or a change in your breasts, where is the first place you would go to get care?	
Tradional healer	25 (2.2%)
Community health clinic	358 (31.7%)
District Hospital	746 (66.1%)

**Fig. 1:**
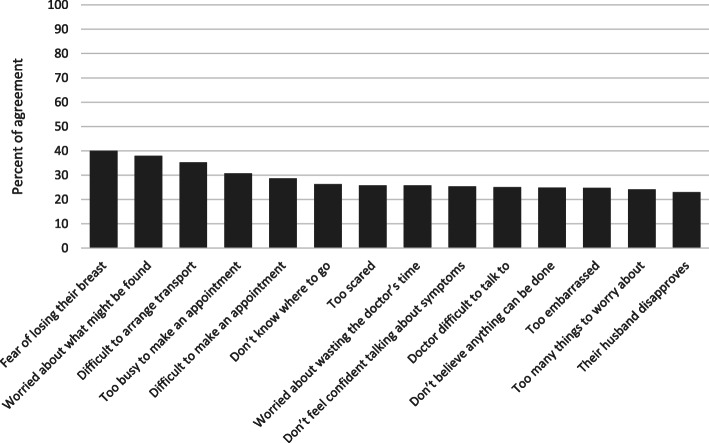
Barriers to care-seeking for breast concerns among community women. Percent agreement from KAP respondents indicating barriers to care seeking. Reponses were chosen from a Likert scale and this graph represents only the “yes, often” responses, which is the highest level of agreement

**Fig. 2:**
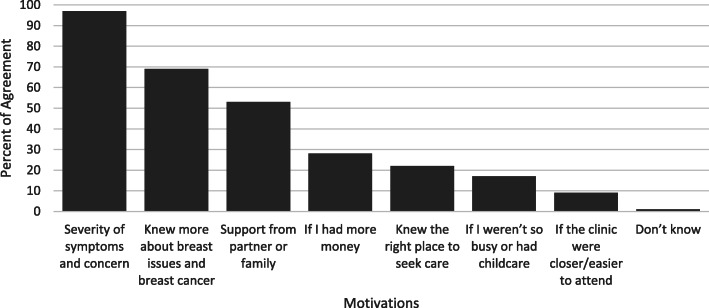
Motivating factors for breast concern care-seeking among KAP participants. KAP respondents were asked if each of these factors would make them more likely to seek care for a breast concern. About 1% of women also stated that “Advice from a Medical Specialist” would be a motivation for care seeking

Of all the assessed barriers to care seeking for breast concerns, women overwhelmingly answered “yes, often” or “yes sometimes” such that the combined responses were at least 95% for each barrier. The three items that women most commonly reported “often” as barriers to care-seeking were fear of losing a breast (40.0%), being worried about what might be found (37.9%), and difficulty arranging transportation (35.3%) **(**Fig. 1**).** The three least chosen barriers were being too embarrassed (24.8%), having too many things to worry about (24.2%), and their husband disapproving (23.0%). On the other hand, the items that women said would motivate them to seek care for a breast concern were severity of the symptoms and concern (97.0%) of women reporting, if they knew more about breast issues and breast cancer (69.0%), and if they had support from their partner or family (53.0%) **(**Fig. 2**)**. Having the clinic closer/easier to attend and if they weren’t so busy or had childcare were least likely to impact their care seeking behaviors.

The average built knowledge score was 24.5% (STD = 0.6) and the self-reported knowledge average was 17.2% (STD = 0.5). The built score increased with age up to age 49 whereas there was no difference in women’s self-reported knowledge by age **(**Table 5**)**. Rural populations comparatively knew less about breast cancer than urban populations as measured by both the built (− 21%) and self-reported (− 4.5%) knowledge scores. There was variability by marital status, and Muslim women tended to have slightly higher built and self-reported knowledge scores compared to Christian women. University educated women scored the highest by both knowledge measures (32.0% built and 28.5% self) but interestingly, women who reported being unsure of highest completed school level or having no schooling scored significantly higher than women with a “class-level” (*P* = 0.011) and “form-level” education (*P* < 0.001) (corresponding to the primary school and middle/high school in the United States respectively) on the built knowledge score. Not surprisingly, women who reported having received prior breast health/cancer education scored higher than women without this education (35.1% vs. 23.2% built and 27.5% vs. 15.9% self). On the other hand, women who have done a breast self-exam had slightly lower knowledge scores compared to women who haven’t done self-exams. In summary, urban dwelling women, those with an occupation classified as profession or manual labor, those with University of higher education, and those with prior breast health education tended to have both the highest built knowledge score (> 30%) and self-reported knowledge (> 20%).

**Table 5:** Factors associated with self-reported knowledge of breast cancer and a built knowledge score based on KAP survey responses

Scores range 0 - 100%		Built knowledge scores Mean 24.5% ± 0.6			Self-reported knowledge scores Mean 17.2% ± 0.5		
		Mean (%)	Comparisons ± STD	*P*-value	Mean (%)	Comparisons ± STD	*P*-value
Age	30-34	20.7	REF		15.7	REF	
	35-39	27.2	6.5 ± 1.8	0.003	18.2	2.5 ± 1.5	0.445
	40-44	29.0	8.2 ± 1.8	0.000	18.4	2.7 ± 1.5	0.395
	45-49	28.5	7.7 ± 2.0	0.001	16.7	1.1 ± 1.7	0.973
	50+	24.3	3.5 ± 1.7	0.231	18.7	3.0 ± 1.4	0.219
Region	Urban	33.1	REF		20.4	REF	
	Peri Urban	30.4	-2.7 ± 1.4	0.121	16.0	-4.4 ± 1.3	0.002
	Rural	11.9	-21.1 ± 1.4	0.000	15.9	-4.5 ± 1.3	0.002
Marriage	Single	22.3	REF		18.6	REF	
	Married	26.3	4.0 ± 1.3	0.008	17.1	-1.4 ± 1.1	0.420
	Divorced/Separated	17.9	-4.4 ± 2.4	0.160	12.0	-6.6 ± 2.0	0.003
Religion	Christian	23.8	REF		16.2	REF	
	Muslim	26.4	2.6 ± 1.4	0.055	19.8	3.6 ± 1.1	0.002
Occupation	Peasant	19.8	REF		18.3	REF	
	Business	23.4	3.6 ± 1.5	0.135	15.1	-3.2 ± 1.3	0.110
	Manual Labor	38.4	18.7 ± 2.5	0.000	26.1	7.8 ± 2.1	0.003
	Professional	34.6	14.8 ± 2.9	0.000	25.9	7.5 ± 2.5	0.022
	Other	23.7	3.9 ± 4.1	0.877	15.4	-2.9 ± 3.5	0.916
Highest Education	No school/Don’t know	29.5	REF		17.6	REF	
	Class 4 or 7/8	23.9	-5.7 ± 1.8	0.011	16.0	-1.5 ± 1.5	0.753
	Form 4 or 6	19.4	-10.1 ± 2.3	0.000	16.4	-1.2 ± 1.9	0.931
	University and Beyond	32.0	2.5 ± 2.8	0.816	28.5	11.0 ± 2.4	0.000
Ever received breast health or breast cancer education	No/Don’t know	23.2	REF		15.9	REF	
	Yes	35.1	11.9 ± 2.0	0.000	27.5	11.6 ± 1.6	0.000
Have you ever heard of cancer	No/Don’t know	14.5	REF		14.4	REF	
	Yes	28.0	13. 6 ± 1.3	0.000	18.1	3.7 ± 1.1	0.001
Family or friend, who has been diagnosed with breast cancer	No/Don’t know	24.7	REF		17.1	REF	
	Yes	23.8	-0.9 ± 1.4	0.498	17.1	-0.0 ± 1.2	0.988
Have you ever done breast self-exam	No/Don’t know	24.9	REF		18.7	REF	
	Yes	23.3	-1.6 ± 1.4	0.271	11.9	-6.8 ± 1.2	0.000

All the numbers in *Table 5* were on percentage scale (0-100%), except for *P* value; STD=standard deviation.

## Discussion

In Tanzania, breast cancer is the second leading cancer in both incidence and mortality among women, with an estimation of 3037 new cases and 1303 deaths in 2018, and is expected to increase to more than 120% for both incidence and mortality by the year 2040 [2, 19]. As a country, Tanzania is relatively new to adopting cancer control programs and investing in community cancer services [3]. This study illustrates the need to factor in women’s perspectives when considering cancer control programs and the strength in conducting knowledge assessments. We did this survey in order to influence future breast cancer control efforts in the lake zone catchment area. In our study, women did not know common risk factors for developing breast cancer such as family history and being older. However, the majority of women did understand that early detection is important to surviving breast cancer, that it is possible to treat breast cancer, and that treatment is available to them in Mwanza. While it is positive that women know breast cancer is treatable, less than one sixth of women indicated they knew any signs or symptoms of breast cancer at all and yet nearly all women would be likely to seek care only if their symptoms were severe. They also indicated numerous barriers they face when care-seeking which is concerning as the National Breast Cancer Control strategy hinges on women recognizing symptoms early and presenting at a health center to obtain a CBE for their breast concern [20]. Other findings had a more positive outlook as over half of women said they would first seek care for breast concerns at a hospital, as compared to a traditional healer, and that they would like to know more about breast cancer. The results of this study demonstrate a clear need for continuing assessments of women’s knowledge and perspectives and offers a starting point for breast cancer educational campaigns to accompany clinical programs for early detection.

Not surprisingly, community women do not have a comprehensive understanding of breast cancer. The majority of women indicated that they had heard of breast cancer and could identify at least breast or armpit lumps as symptoms but very few women had received any breast cancer education previously. This is not uncommon in East Africa, with surveys in coastal and Western Kenya reporting similar results [21, 22]. In another Tanzanian study based in Dar es Salaam, 70.2% of women looking for outpatient care at the district hospital recognized breast lump successfully [23]. In a survey among 230 Nigerian women, only 50% of the women noted a painless lump as the breast cancer symptom [24]. Similarly, in a large survey assessing the perceptions of breast cancer among non-healthcare personnel in Kenya only around 12% recognized breast lump as a symptom and less than 2% recognized breast rash, and changes in the nipple as signs and symptoms [21]. This is also true of risk factor knowledge. Of the women surveyed, very few agreed that being older or having a close relative with breast cancer can increase the chance of getting breast cancer. In a study from Western Kenya, 52.5% of those surveyed did not know any breast cancer risk factors and only 12.3% mentioned heredity [21]. Comparatively, in the study of Nigerian women, around 45 and 33% thought family history and old age were breast cancer risk factors, respectively [24]. For cancer control programs to be successful at detecting breast cancer early, especially programs that rely on women taking the initiative to seek care, community women must possess the bare minimum knowledge of breast cancer signs, symptoms and risk factors.

General barriers to care seeking specifically for a breast concern were overwhelming. Of each presented barrier, fear of losing their breast was the common response. This sentiment is likely reflective of the current method of treatment, as surgery is generally the first and most common procedure, and breast-conserving/breast-sparing options are nascent in many LMIC settings [2, 25]. Similar research has explored the idea of prioritizing responsibility for other family members needs over the woman’s own [26, 27] and this is also reflective in our research as majority of women were worried about what might be found in a medical exam and only present when their symptoms are severe and presumably interfere with everyday life. Studies have acknowledged that low awareness of breast cancer symptoms may prevent women from noticing, or even misinterpret and ignore these symptoms, which contribute to the delay of women’s presentation in the hospital and subsequent diagnosis [21, 28]. For example, when women had detected lumps, they may still wait until the appearance of other symptoms, such as pain, before turning to medical help [21]. Additionally, when considering women with only non-lump symptoms, studies have shown an even greater delay in seeking care compared to those women who present with lump only or lump plus additional symptoms. Arranging transportation was also a commonly reported barrier to care seeking, which was also reflected in the low reported ownership of transportation methods and has been shown to be an issue for accessing care LMICs [29, 30]. However, almost all women in our study indicated that they would be motivated to seek care if their symptoms were severe. This is worrisome that severity of symptoms is a motivator since it is the opposite of cancer controls “early detection” goal. Late presentation, diagnosis, and treatment have been serious challenges for breast cancer control in many other LMICs, but elevated breast cancer awareness has shown to be associated with higher breast self-examination practice frequency, screening participation, earlier presentation, and better follow-up to referral and treatment [9, 16, 26, 27, 31–34].

Looking at overall knowledge, women do not believe they know a lot about breast cancer and did not demonstrate high health awareness of breast cancer. Groups that typically knew less about breast cancer were from rural areas, tended to be younger and identified their occupation as ‘peasant’. Our study revealed that previous health related education or university level education, were both factors for high built knowledge scores. However, the level of education received did not affect women’s breast cancer awareness significantly. This finding was consistent with a study in China [35], but deviates from other studies in Nigeria [36], Pakistan [37], and Saudi Arabia [38], which reported a potentially positive relationship between education level and breast cancer awareness. Several retrospective studies have shown the importance of breast health awareness and early presentation as they positively impact survival [39–41]. When developing health education programs it is important to target groups that both need education and can share and disseminate knowledge to peers and other community members [42, 43]. Religious outreach campaigns can utilize the community aspect of religion to target and recruit participants [44]. In addition, several studies have acknowledged the impact of religion on breast cancer control, and suggest incorporating a component of religion when developing health education programs [45, 46]. Educational campaigns at schools similarly can easily recruit participants and the knowledge learned while young will follow a woman through her life arming her with the necessary tools to recognize suspicious symptoms and seek care sooner [47].

Moving forward, this initial study gives clear guidance on where basic gaps exist regarding community knowledge and barriers to care. Strengths of this study include the large sample size and distribution of women all over Mwanza. Our population was also not limited to women already in a healthcare setting. We sampled women from public areas where people congregate, and this made our study population more representative of the general population and our results generalizable for the lake region. However, our use of convenience sampling had limitations, including a large sample of 30–39 year old women while only capturing the perspectives of few women over the age of 60. In Tanzania, this age category accounts for about 5% of the total population whereas they only comprised 3.4% of our study sample [48]. Finally, the KAP survey was based on a well validated and widely used instrument to assess breast cancer awareness, ensuring comparability with other studies, although, adaptations were needed to best fit the Tanzanian study context. As this is a good initial assessment of knowledge and practices, further assessments into care seeking and beliefs are necessary to explore the topics of care seeking motivations and barriers. In-depth qualitative assessments would allow us to better characterize true barriers to knowledge and care-seeking and develop targeted, evidence-informed interventions.

## Conclusions

The Tanzania guidelines hinge on women seeking care for breast concerns. When women are delayed or do not understand the significance of their symptoms they will present later, which limits treatment options and decreases breast cancer survival. These shortfalls in basic breast cancer facts can be targets for future education campaigns and can point to informing dissemination strategies. Our study found that women believed if they knew more about breast health and breast cancer, they would be more motivated to seek care for breast concerns and they also indicated they would seek care in a hospital setting first for these concerns. This finding illustrates the community’s desire for more knowledge and sets up future campaigns for success, as women should be more receptive to learning. The Tanzanian National guidelines focused on CBE should be coupled with clear dissemination strategies that include community awareness and education of women to promote care-seeking. At the current capacity, breast cancer treatment is most effective for women who present early and this would ensure program success. As our study shows, there are still a multitude of barriers that women face when pressed with care seeking and the top two indicated barriers deal with fear of the diagnosis and treatment itself. Education focused on the whole cancer care continuum are imperative for women to understand the outcomes of early presentation as they compare to late presentation and the availability of treatment to her. Community involvement is critical to successful cancer control. As Tanzania and other countries continue to build their cancer treatment capacity, so should they build up their outreach and community education capacity.

## Data Availability

The dataset analyzed in the current study is not publicly available due to ongoing analysis and manuscript creation but requests for research collaboration using the data may be submitted to the corresponding author.

## Abbreviations

LMIC: Low- and middle- income countries
HIC: High-income countries
BMC: Bugando Medical Centre
KAP: Knowledge, Attitudes and Practices
NIMR: National Institutes for Medical Research, Tanzania
CUHAS – BMC: Catholic University of Health and Allied Sciences-Bugando Medical Centre
JHSPH: Johns Hopkins Bloomberg School of Public Health
BCAM: Breast Cancer Awareness Measures
ANOVA: Analysis of Variance
BMI: Body Mass Index
CBE: Clinical Breast Exam
